# Label-free method to monitor metabolism during long-term culture of human pluripotent stem cell derived cardiomyocytes

**DOI:** 10.1117/1.BIOS.2.2.025001

**Published:** 2025-04-17

**Authors:** Tongcheng Qian, Danielle E. Desa, Emmanuel Contreras Guzman, Wenxuan Zhao, Xiaotian Zhang, Sean P. Palecek, Melissa C. Skala

**Affiliations:** a Morgridge Institute for Research, Madison, Wisconsin, United States; b University of Wisconsin–Madison, Department of Biomedical Engineering, Madison, Wisconsin, United States; c University of Wisconsin–Madison, Department of Chemical and Biological Engineering, Madison, Wisconsin, United States

**Keywords:** stem cell, cardiomyocyte, maturation, autofluorescence, label-free, optical metabolic imaging

## Abstract

**Significance:**

Human pluripotent stem-cell–derived cardiomyocytes (hPSC-CMs) are a powerful tool for drug discovery, and metabolic changes are associated with their long-term culture and maturation. However, the lack of technologies to monitor hPSC-derived cardiomyocyte metabolism during long-term culture presents a major technical bottleneck.

**Aim:**

Efforts to monitor *in vitro* metabolic maturation of hPSC-CMs are limited by traditional assessment methods, which are generally time-consuming, destructive to samples, and lack single-cell resolution. We report a rapid, noninvasive imaging-based method to monitor hPSC-CM metabolism throughout extended culture (90+ days).

**Approach:**

Label-free optical metabolic imaging (OMI) of autofluorescent metabolic coenzymes was performed at multiple time points during the extended culture maturation process. In addition, OMI monitored hPSC-CMs grown on substrates with varying stiffness and on cardiomyocytes derived from induced pluripotent stem cells associated with cardiac arrhythmia. OMI was paired with immunofluorescence to validate structural maturation.

**Results:**

Single-cell OMI can identify metabolic changes during cardiomyocyte maturation through extended *in vitro* culturing. It can also detect metabolic differences induced by substrates of varying stiffnesses, can distinguish diseased from normal cell lines, and is sensitive to patient-level metabolic heterogeneity.

**Conclusions:**

Our results demonstrate that label-free OMI can be used to monitor metabolic changes in hPSC-CMs under varying culture conditions in a rapid, non-destructive manner with single-cell resolution, providing insight into metabolic transitions arising from time in culture, culture conditions, or disease states.

## 1 Introduction

Cardiovascular disease (CVD) remains the leading cause of death worldwide despite advances in treatment.^1^ CVD causes over 30% of American deaths and accounts for 14% of total US healthcare costs.^2^ Human pluripotent stem-cell–derived cardiomyocytes (hPSC-CMs) have immense potential to impact fundamental research and clinical care for CVD, enabling better disease modeling, drug screening platforms, and tissue regeneration options.^3–5^ Different methods of efficiently generating cardiomyocytes from hPSCs have been well established, resulting in batches with >80% purity.^6,7^ Maturation of these cells requires coordination and crosstalk among regulatory pathways to produce fully functional cardiomyocytes^8^ that can be used for different purposes at various maturation stages.^9^ For example, cardiac progenitor cells can be used for modeling development and for cell-based therapies,^10–12^ whereas mature cells have been used to model CVD (e.g., inherited arrhythmia),^13,14^ study drug toxicity,^15,16^ and generate organ-like tissues.^17–20^ However, hPSC-CMs fail to fully mature *in vitro*, significantly impeding their utility in CVD research and clinical translation.^21,22^

Standard methods to assess facets of cardiomyocyte maturation rely on analysis of cell structure (e.g., immunofluorescence to visualize sarcomere alignment and spacing),^23^ gene expression (e.g., RNA sequencing to monitor myosin and troponin isoform switching),^9,24^ metabolic state (e.g., oxygen consumption measurements),^23,25^ and electromechanical function (e.g., electrophysiology and contractile force generation).^9^ These techniques are also used to evaluate the effectiveness of strategies to enhance *in vitro* maturation.^26^ Although most of these standard techniques are accurate and specific, they disrupt the cells, are time-consuming, and/or do not offer single-cell resolution. This single-cell resolution is critical because heterogeneity is routinely observed during long-term hPSC-CM culture as not all CMs mature in synchrony.^27–29^ Therefore, noninvasive technologies that can repeatedly monitor hPSC-CM cultures at the single-cell level are needed to improve hPSC-CM production.

Throughout the maturation process, cardiomyocyte metabolism changes substantially.^30^ Fetal cardiomyocytes rely heavily on glycolysis for energy production to support macromolecule biosynthesis and cell proliferation in a low-oxygen environment. At the neonatal stage and beyond, mitochondrial oxidative phosphorylation dominates adenosine triphosphate production, with fatty acids as the main substrate.^31^ Nicotinamide adenine dinucleotide (phosphate) (NAD(P)H) and flavin adenine dinucleotide (FAD) are coenzymes involved in metabolism that intrinsically fluoresce within cells in their reduced and oxidized states, respectively.^32,33^ Optical metabolic imaging (OMI) is a powerful tool capable of noninvasively collecting autofluorescence lifetimes from NAD(P)H and FAD at the single-cell level.^32,34,35^ The fluorescence lifetime measures how long a molecule remains excited before emitting a photon and changes based on NAD(P)H or FAD protein-binding activity as well as fluctuations in environmental pH, viscosity, and temperature.^35–38^ Previously, OMI has been used to predict the efficiency of induced pluripotent stem cell (iPSC)-CM batch differentiation within the first 24 h of differentiation,^39^ distinguish iPSCs differentiating into dermal fibroblasts versus keratinocyte progenitor cells,^40^ and track mesenchymal stem cell differentiation into adipocytes,^41,42^ osteocytes,^42–44^ and chondrocytes.^43^ OMI has also been used at the tissue level to monitor cartilage maturation and degeneration^45^ and track long-term maturation in stem cell–derived cultures.^46,47^ Critically, OMI is repeatable and live cell compatible, allowing longitudinal tracking of samples and enabling the performance of traditional endpoint analyses on the same cell batches.

Here, we demonstrate that OMI can be used to monitor metabolic changes of hPSC-CMs during maturation in extended culture. Flow cytometry and immunofluorescence were used to quantify cardiomyocyte purity and verify structural maturation, respectively. Cardiomyocytes derived from three hPSC lines were imaged from early (day 30) through late stage (day 90 to 120) maturation. Lines tested include an embryonic stem cell (ESC) line (H9) and iPSC lines with disease phenotypes (long QT syndrome, also known as cardiac arrhythmia) to demonstrate the versatility of OMI across lines. Changes in OMI variables reflected expected metabolic shifts that correspond to morphological changes observed during cardiomyocyte maturation. OMI also effectively monitored substrate-induced metabolic changes in hPSC-CMs seeded on stiffnesses ranging from 1.5 kPa to 1 GPa, reflecting the influence of the mechanical environment on cardiomyocyte metabolism. Finally, OMI changes over time differed between the ESC and iPSC lines with long QT syndrome, indicating variable metabolic trajectories between healthy hPSC-CMs and diseased iPSC-CMs. These results support OMI as a noninvasive real-time method to monitor single-cell metabolism during hPSC-CM manufacturing. More broadly, this label-free technology can be applied to both quality control of cardiomyocyte biomanufacturing and the study of cardiac disease mechanisms.

## 2 Methods

### 2.1 hPSC Culture, Cardiomyocyte Differentiation, and Maturation

Human H9 ESCs^48^ and iPSCs linked to long QT syndrome (UCSD 102i-2-1 and UCSD 106i-2-5)^49^ were cultured on Matrigel (Corning Inc., Corning, New York, United States)-coated surfaces in mTeSR1 (STEMCELL Technologies Inc., Cambridge, Massachusetts, United States) as previously described.^7^ Cardiomyocyte differentiation was performed as described previously.^7^ Briefly, hPSCs were singularized with Accutase (ThermoFisher Scientific, Waltham, Massachusetts, United States) and plated on Matrigel-coated plates at a density of ~2 × 10^5^ cells∕cm^2^ (7 × 10^5^ cells∕well of a 12-well plate) in mTeSR1 supplemented with 10 *μ*M Rho-associated protein kinase inhibitor Y-27632 (Selleck Chemicals LLC, Houston, Texas, United States) 2 days prior to differentiation. Differentiation was initiated by Wnt signaling activation with 8 *μ*M CHIR99021 (Selleck Chemicals) on day 0, followed by inhibition of Wnt signaling with 5 *μ*M IPW2 on day 3. On day 5, the medium was switched to RPMI 1640 (Gibco, ThermoFisher) supplemented with 2% B27 minus insulin (ThermoFisher), followed by subsequent replacements with RPMI 1640 with 2% B27 every other day starting from day 7. By day 12, fully differentiated cardiomyocytes were dissociated using Accutase and reseeded onto Matrigel-coated imaging dishes or Matrigel-coated polydimethylsiloxane (PDMS) with different stiffnesses (1.5, 15, and 28 kPa, purchased from Ibidi USA Inc., Fitchburg, Wisconsin, United States) at a density of 1 × 10^5^ cells∕cm^2^, and medium was replaced every 3 days.^7^

### 2.2 Flow Cytometry

Cardiomyocytes on differentiation days 20 and 40 were disassociated with Accutase, fixed in ice-cold 1% PFA for 15 min, and then blocked with 0.5% bovine serum albumin (BSA) with 0.1% Triton X-100. Cells were then stained with primary antibody anti-MF20 (Mouse IgG2b/DSHB; 1:20) and Ki67 (Mouse IgG1/BD Biosciences; 1:100) secondary antibody (ThermoFisher; goat anti-mouse Alexa 488 goat anti-mouse IgG1/A-21121, Alexa 647 goat anti-mouse IgG2b/A-21242; 1:500) in 0.5% BSA with 0.1% Triton X-100. Data were collected on a FACSCalibur flow cytometer (BD Biosciences, Franklin Lakes, New Jersey, United States) and analyzed with FlowJo (BD Biosciences). Data were collected from three biological replicates and presented as mean ± standard error of the mean (SEM). MF20 and Ki67 positive percentages were rounded up one decimal place.

### 2.3 Autofluorescence Imaging of NAD(P)H and FAD

Fluorescence lifetime imaging (FLIM) was performed using an Ultima two-photon imaging system (Bruker, Madison, Wisconsin, United States) composed of an ultrafast tunable laser source (Insight DS+, Spectra Physics, Santa Clara, California, United States) coupled to a Nikon Ti-E inverted microscope (Tokyo, Japan) with time-correlated single-photon counting electronics (SPC-150, Becker & Hickl GmbH, Berlin, Germany). The ultrafast tunable laser source enables sequential excitation of NAD(P)H at 750 nm and FAD at 890 nm. NAD(P)H and FAD emissions were isolated using 440/80 and 550/100 nm bandpass filters (Chroma, Bellows Falls, Vermont, United States), respectively. The laser power at the sample for NAD(P)H and FAD excitation was ~6.5 and 15 mW, respectively. Fluorescence lifetime decays with 256-time bins were acquired across 256 × 256 pixel images with a pixel dwell time of 4.8 *μ*s and an integration period of 60 s and collected using GaAsP photomultiplier tubes (H7422, Hamamatsu, Shizuoka, Japan). All samples were illuminated through a 40×/1.15 NA objective (Nikon). FLIM was performed on cardiomyocytes in a stage-top incubator at 37°C and 5% CO_2_ (Tokai Hit USA, Inc., Bala Cynwyd, Pennsylvania, United States). The instrument response function (full width at half maximum ~260 ps) was acquired from the second harmonic generated signal of urea crystals at 890 nm and was measured for each imaging session.

### 2.4 Image Analysis

Lifetime images of NAD(P)H and FAD were analyzed via SPCImage software (Becker & Hickl), as described previously.^39^ Briefly, two-component decays were calculated by the following equation: $I\left(t\right)=\alpha_{1}e^{-t/\tau_{1}}+\alpha_{2}e^{-t/\tau_{2}}+C$^37^ where *I*(*t*) is intensity as a function of time, *τ*_1_ is the short lifetime, *τ*_2_ is the long lifetime, *α*_1_ and *α*_2_ are the relative proportions of the short and long lifetimes, respectively, and *C* is background light. Fluorescence intensity images were generated by integrating photon counts over the per-pixel fluorescence decays. For FLIM analysis of cardiomyocytes, pixels were binned to 1 (3 × 3 pixels) to achieve sufficient statistics for fluorescence decay fitting. Single-cell segmentation was performed using custom Cellpose models trained on manually annotated cardiomyocytes (whole cell and nuclei, training *n* = 14, test *n* = 6).^50,51^ Whole-cell and nuclear masks were manually corrected as needed using the napari viewer. Cytoplasm masks were generated by removing nuclear from whole-cell masks and applied to all images to determine single-cell NAD(P)H and FAD fluorescence lifetime variables. Fluorescence lifetime variables consist of the mean lifetime (*τ*_*m*_ = *α*_1_*τ*_1_ + *α*_2_*τ*_2_), free-bound and protein-bound lifetime components (*τ*_1_ and *τ*_2_ for NAD(P)H and *τ*_2_ and *τ*_1_ for FAD, respectively), and their fractional contributions (*α*_1_ and *α*_1_, where *α*_1_ + *α*_2_ = 1) for each cell cytoplasm.^34–36^

### 2.5 Classifier Methods

A random forest classifier was trained in Python using scikit-learn to classify healthy and diseased cells using eight OMI variables (NAD(P)H *τ*_*m*_ *τ*_1_, *τ*_2_, *α*_1_; FAD *τ*_*m*_ *τ*_1_, *τ*_2_, *α*_1_). The classifier was trained on a random selection of 70% of the single-cell data (*N* = 1564 H9 and *N* = 3493 long QT cells) and tested on the remaining 30% (*N* = 671 H9 and *N* = 1497 long QT cells). We used the following configuration of the random forest model: number of trees in the forest = 100; no maximum depth of each tree; minimum number of samples required to split an internal node = 2.

Multiple metrics were used to evaluate the robustness of the classifier, including the calculated area under the curve of the receiver operating characteristic (AUC of ROC) as well as accuracy, precision, and recall from the confusion matrix of the random forest classifier of the test data. All feature importance values add up to 1.

### 2.6 Dimensionality Reduction and Visualization

Uniform manifold approximation and projection (UMAP) dimensionality reduction was performed on eight OMI variables (NAD(P)H and FAD *τ*_*m*_, *τ*_1_, *τ*_2_, *α*_1_) for projection in two-dimensional space to visualize clustering in the imaging datasets. The following parameters were used for UMAP visualizations: “n _neighbors”: 15, “min_dist”: 0.1, “metric”: cosine, “n_components”: 2.

### 2.7 Statistics

OMI variables are presented as mean ± SD (Figs. 2, 3, and 5, and Figs. S1, S3, S5, and S6 in the Supplementary Material). Flow cytometry data are presented as mean ± SEM. Statistical significance was determined by an unpaired two-tailed *t*-test with *p* < 0.05 considered statistically significant. The effect size was determined by Glass’s Δ, with <0.2 considered a small effect size, 0.2 to 0.5 medium, 0.5 to 0.8 large, and >0.8 very large.

## 3 Results

### 3.1 Optical Metabolic Imaging Detects Changes in NAD(P)H and FAD Autofluorescence During Cardiomyocyte Maturation

**Fig. 1:**
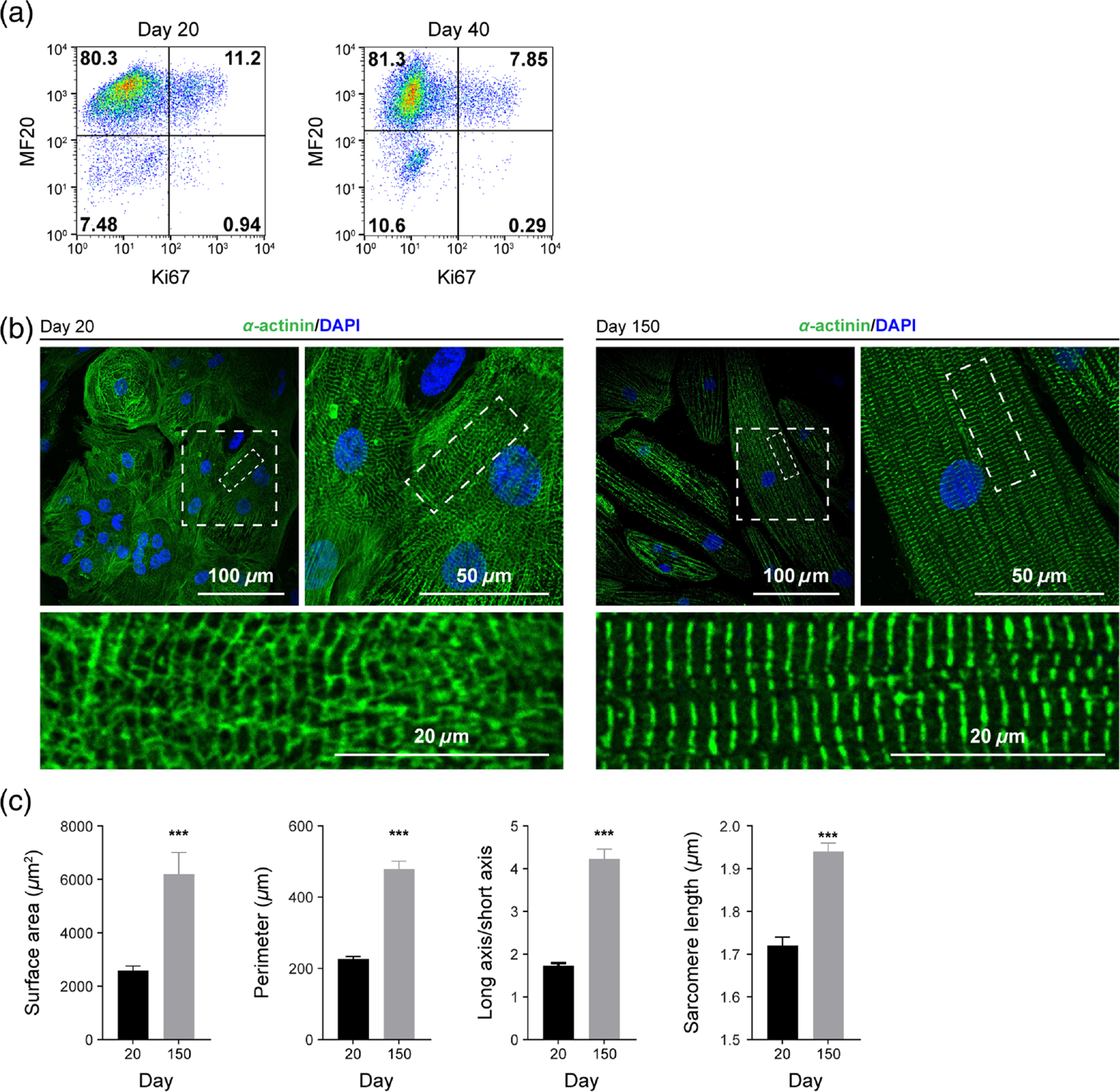
hPSC-derived cardiomyocytes exhibit more mature structural and proliferative phenotypes with extended *in vitro* culturing. H9 ESC-derived cardiomyocyte differentiation and cell proliferation were confirmed by flow cytometry for heavy chain myosin II (MF20 antibody) and Ki67 expression on days 20 and 40, respectively. (a) Representative flow cytometry dot plots with a gating strategy to determine the percentage of MF20 and Ki67-positive cells. Day 20, 12.2 ± 1.6% Ki67+; day 40, 8.8 ± 1.4% Ki67+. (b) Representative immunofluorescent *α*-actinin staining after early differentiation (day 20, left) and extended culture (day 150, right). (c) Single-cell size, elongation, and average sarcomere length per cell were quantified on day 20 (*N* = 27, three independent experiments) and day 150 cardiomyocytes (*N* = 22, three independent experiments). Data are presented as mean ± SD. Statistical significance was determined by an unpaired two-tailed *t*-test, ***p < 0.001.

Cardiac metabolism changes throughout heart development and cardiomyocyte maturation.^25,31,52^ During the maturation process, cardiomyocyte metabolism shifts from primarily relying on glycolysis to heavily depending on oxidative pathways.^31^ NAD(P)H regulates cellular metabolism and redox state^53^; thus, we predict its autofluorescence properties can be used to noninvasively monitor cardiomyocyte metabolism.^39^ To test this notion, we cultured hPSC-CMs *in vitro* and observed the maturation process over 150 days using multiple analysis methods. Over 85% of the cell population was first verified as cardiomyocytes by flow cytometry labeling for the cardiac-specific protein, sarcomeric myosin heavy chain [MF20 antibody, Fig. 1(a)].^54^ To confirm the change in sarcomere organization and length during maturation, hPSC-CMs were characterized by immunofluorescence labeling with *α*-actinin [Fig. 1(b)], and cell proliferation was quantified by flow cytometry for Ki67 [Fig. 1(a)]. hPSC-CMs exhibited low proliferative activity with 12.2% (±1.6%) Ki67-positive population at an early stage (day 20), which dropped to 8.8% (±1.4%) by day 40, as expected [Fig. 1(a)]. *α*-Actinin was labeled, and DAPI was used as a counterstain to characterize structural changes between days 20 and 150, including increased cell surface area, cell perimeter, the ratio of long versus short cell axes, and individual sarcomere length. Compared with day 20, these morphological parameters all significantly increased in CMs on day 150, consistent with previous studies^23^ [Figs. 1(b) and 1(c)].

OMI was then performed on H9 ESC-CMs on days 30, 40, 60, 70, and 90 to monitor longterm *in vitro* metabolic maturation. A total of eight OMI variables were calculated at the single-cell level: NAD(P)H lifetime fit parameters (*τ*_*m*_*, τ*_1_*, τ*_2_*, α*_2_), and FAD lifetime fit parameters (*τ*_*m*_*, τ*_1_*, τ*_2_*, α*_1_). The short lifetime (*τ*_1_) corresponds to free NAD(P)H, whereas the long lifetime (*τ*_2_) corresponds to protein-bound NAD(P)H. The converse applies to FAD *τ*_1_ (protein-bound) and *τ*_2_ (free).^37,55^ Weights are applied to the short (*α*_1_) and long (*α*_2_) lifetimes, and the mean lifetime is a weighted average (*τ*_*m*_ = *α*_1_*τ*_1_ + *α*_2_*τ*_2_).

**Fig. 2:**
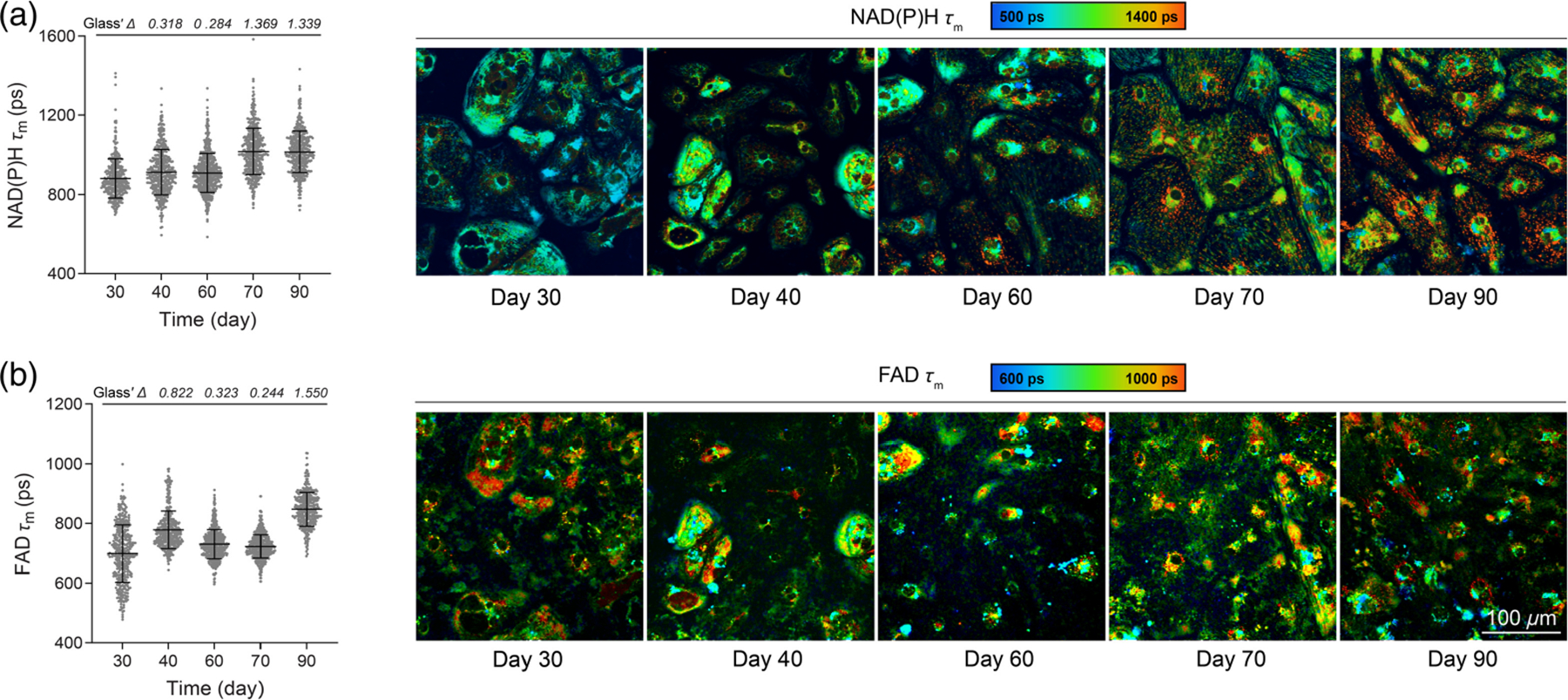
OMI detects fluorescence lifetime changes of NAD(P)H and FAD during long-term *in vitro* maturation. Multiphoton autofluorescence OMI was performed on H9 ESC-derived cardiomyocytes throughout extended maturation between days 30 and 90. Single-cell cytoplasmic quantitative analysis of (a) NAD(P)H mean lifetimes (*τ*_*m*_) and (b) FAD mean lifetimes (*τ*_*m*_). Corresponding representative images are pseudocolored by mean lifetime and show the subcellular distribution of metabolites. Punctate, low lifetime features are seen in the lower images, potentially from lipid droplets that emit broadly within the same range as FAD. Cells were imaged on days 30, 40, 60, 70, and 90 (*N* = 415, 485, 582, 465, 380 cells, respectively, 10 fields of view from three independent experiments). Data are presented as mean ± SD. The effect size was determined using Glass’ Δ with >0.80 considered a very large effect.

NAD(P)H mean lifetime (*τ*_*m*_) increased from early (day 30) to late maturation (day 90) [Fig. 2(a)], with the largest changes seen by days 70 and 90. This is due to an increase in the free and bound lifetimes of NAD(P)H (*τ*_1_ and *τ*_2_) and an increase in the proportion of bound NAD(P)H (*α*_2_) [Figs. S1(a)–S1(c) in the Supplementary Material]. FAD mean lifetime (*τ*_*m*_) increased from early (day 30) to late maturation (day 90). However, it showed a slight decrease on days 60 and 70 compared with day 40 [Fig. 2(b)] primarily due to the changes in the bound lifetime of FAD (*τ*_1_) and the proportion of bound FAD (*α*_1_) [Figs. S1(d)–S1(f) in the Supplementary Material]. Single-cell histograms showed that cardiomyocyte populations exhibited relatively homogenous NAD(P)H *τ*_*m*_ and FAD *τ*_*m*_ distributions for days 40, 60, and 70. By contrast, NAD(P)H *τ*_*m*_ exhibited a bimodal distribution on 90, whereas FAD *τ*_*m*_ exhibited a bimodal distribution on day 30 (Fig. S2 in the Supplementary Material), indicating metabolic heterogeneity during early and late maturation at a later stage within the culture.

### 3.2 Optical Metabolic Imaging Monitors Changes in Cardiomyocytes Over Time on Substrates with Varying Stiffness

Cardiomyocytes experience various mechanical forces *in vivo*, including stiffening of the heart extracellular matrix during development.^56^ These mechanical cues are critical to heart development, with cardiac matrix stiffening from <0.2 kPa at the formation of the cardiac tube to 20 kPa in human neonates.^56^ Alteration of mechanical force can also drastically impact cardiomyocyte maturation,^57–59^ and these forces are recapitulated in part through the stiffness of the culture substrate *in vitro*. In the adult heart, the physiological stiffness of the extracellular matrix (ECM) is ~10 kPa.^60^ Substrates with stiffnesses of 10 to 20 kPa have been shown to improve cardiomyocyte maturation by increasing cell size, sarcomere length, and protein expression related to maturation.^61^ We detected changes in NAD(P)H and FAD lifetime fit parameters during maturation on Matrigel-coated plates (Fig. 2), so we further investigated if OMI could detect changes in CM metabolism over time on substrates of varying stiffness.

**Fig. 3:**
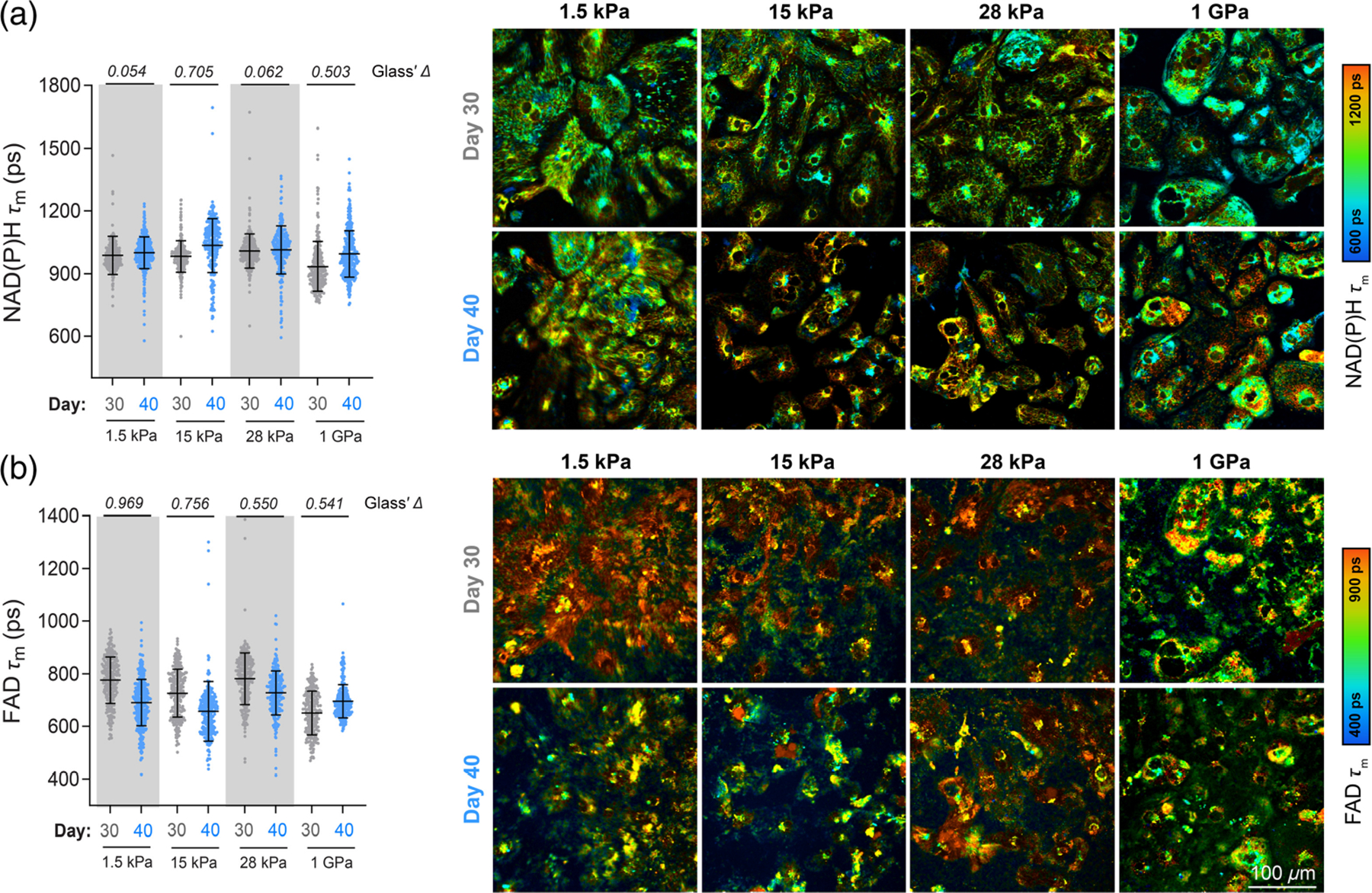
OMI monitors metabolic changes over time on substrates with varying stiffness. H9 ESC-derived cardiomyocytes were cultured on commercial Matrigel-coated PDMS surfaces with different stiffnesses (1.5, 15, and 28 kPa) or Matrigel-coated polymer-bottomed dishes (1 GPa), and multiphoton FLIM was performed during early maturation (days 30 and 40). Single-cell cytoplasmic analysis of (a) NAD(P)H mean lifetimes (*τ*_*m*_) and (b) FAD mean lifetimes (*τ*_*m*_) with corresponding representative images, respectively. Cells were imaged on day 30 (*N* = 271, 289, 269, and 274 cells, with increasing stiffness, 10 fields of view from three independent repeats) and day 40 (*N* = 395, 295, 267, and 375 cells, with increasing stiffness, 10 fields of view from three independent repeats). Data are presented as mean ± SD. Effect size was calculated using Glass’ Δ, with >0.5 considered a large effect and with >0.8 considered a very large effect.

H9 ESC-CMs were replated on Matrigel-coated PDMS surfaces with different stiffnesses (1.5, 15, and 28 kPa) and Matrigel-coated polymer-bottomed dishes (~1 GPa)^62^ on day 12. Long-term culture of adherent cells on ECM-coated PDMS results in cell clustering and subsequent dissociation,^63^ and according to previous studies, day 20 to day 30 PSC-CMs reflect the late embryonic stage.^64^ Therefore, imaging was only performed during early maturation. We measured NAD(P)H *τ*_*m*_ and FAD *τ*_*m*_ on days 30 and 40 and quantified the effect size (Glass’s Δ) for each stiffness. As shown in Fig. 3(a), the greatest increase in NAD(P)H *τ*_*m*_ between days 30 and 40 was observed in cells plated on 15 kPa substrate (Glass’s Δ = 0.705), whereas minimal differences were seen on the softer (1.5 kPa, Glass’s Δ = 0.054) and the stiffer substrates (28 kPa, Glass’s Δ = 0.062). Changes in NAD(P)H *τ*_*m*_ at 15 kPa were mainly due to increases in bound NAD(P)H [*τ*_2_, Figs. S3(a)–S3(c) in the Supplementary Material] at day 40 relative to day 30. The FAD *τ*_*m*_ between days 30 and 40 changed the most for cells on the softest substrate (1.5 kPa, Glass’s Δ = 0.969). The magnitude of change decreased with increasing stiffness [Fig. 3(b), Glass’s Δ = 0.756, 0.550, 0.541 at 15 kPa, 28 kPa, 1 GPa, respectively] driven mainly by changes in protein-bound FAD [*τ*_1_, *α*_1_, Figs. S3(d)–S3(f) in the Supplementary Material]. Representative images (Fig. 3) demonstrate that OMI can be used to noninvasively monitor metabolic changes occurring in cells maturing on substrates of various stiffness.

### 3.3 Optical Metabolic Imaging Detects Differences in Cardiomyocyte Maturation of Diseased Phenotypes

Inherited and acquired CVDs are partly attributed to metabolic dysfunction that results from altered signaling and genetic expression.^65,66^ An important example is long QT syndrome, a potentially life-threatening arrhythmia caused by genetic mutations that disrupt ion currents and subsequent repolarization of the heart.^67^ Prolonged long QT interval is a comorbidity seen increasingly with metabolic disorders, including hypoglycemia and diabetes.^68,69^ We investigated if OMI could monitor the extended culture maturation process of CMs derived from iPSCs with long QT syndrome as iPSCs can carry genetic mutations from parental somatic cells and therefore exhibit disease phenotypes in differentiated cell lineages.^49,70^ The two lines used here—iPSC UCSD 102i-2–1, from an 18-year-old Caucasian female, and iPSC UCSD 106i-2–5, from a 45-year-old Asian/Caucasian female^49^—both carry the p.W1001* mutation in *KCNH2*. This mutation encodes the *α* subunit of a potassium ion channel crucial for the final repolarization of the ventricular action potential and leads to long QT syndrome 2.^71^

**Fig. 4:**
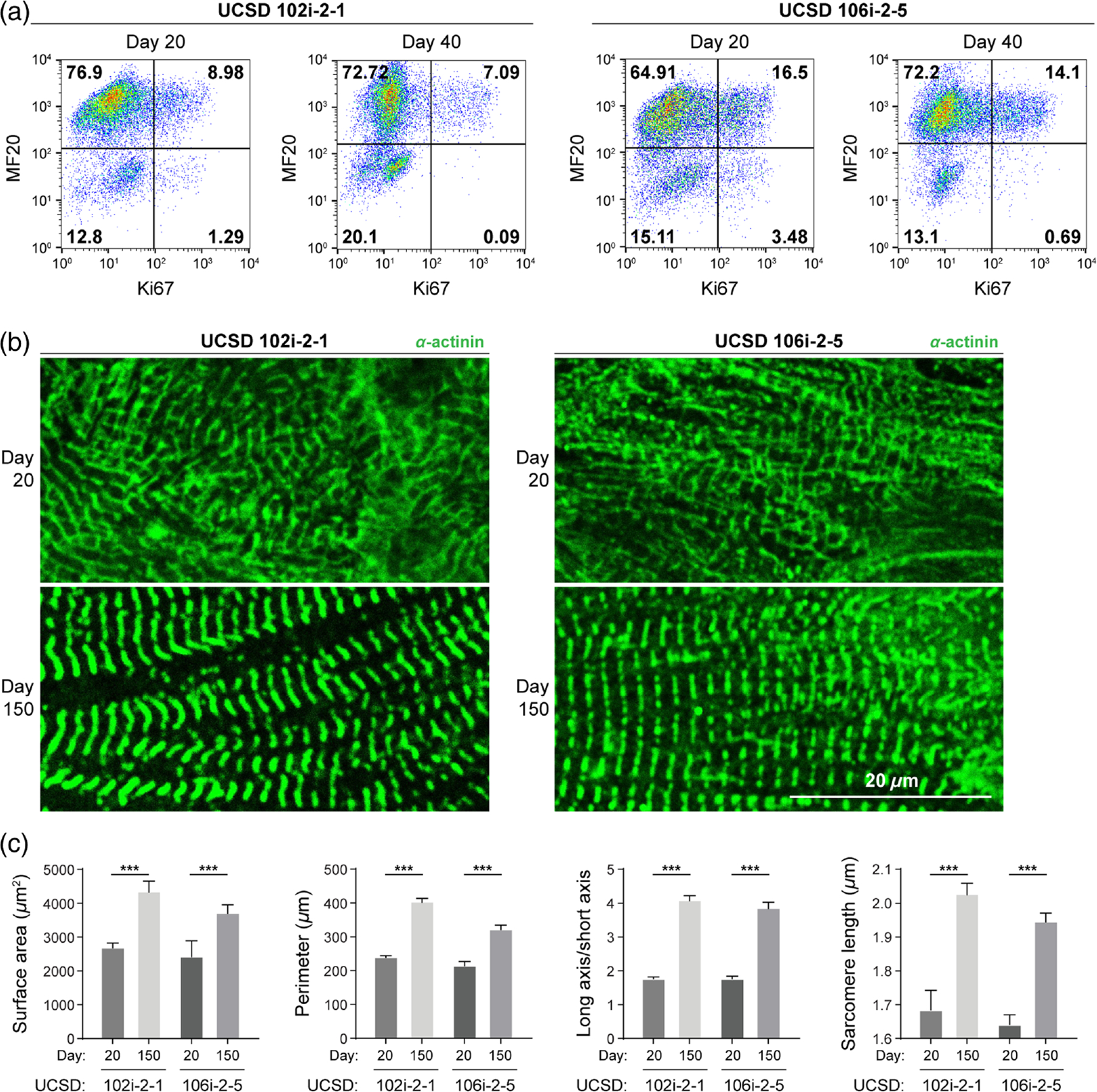
iPSC-derived long QT cardiomyocytes exhibit a mature phenotype with extended *in vitro* culturing. Human iPSCs carrying a long QT syndrome genetic deficiency were differentiated into cardiomyocytes (UCSD102i-2–1, UCSD106i-2–5, from separate donors, respectively) following an established method. Differentiation was confirmed by flow cytometry for heavy chain myosin II (MF20) and Ki67 expression on days 20 and 40. (a) Representative flow cytometry dot plots with a gating strategy to determine the percentage of MF20 and Ki67-positive cells. UCSD102: day 20, Ki67+ 10.5%; day 40, Ki67+ 8.9%. UCSD106: day 20, Ki67+ 20.3%; day 40, Ki67+ 16.7%. 40. (b) Representative immunofluorescent *α*-actinin staining after early differentiation (day 20) and extended culture (day 150). (c) Single-cell size, elongation, and average sarcomere length per cell were quantified in newly differentiated cardiomyocytes (day 20, UCSD102i-2–1, *N* = 27, from three independent repeats; UCSD106i-2–5, *N* = 24, from three independent repeats) and after extended culture (day 150, UCSD102i-2–1, *N* = 45, from 3 independent repeats; UCSD106i-25, *N* = 50, from 3 independent repeats). Data are presented as mean ± SD. Statistical significance was determined by an unpaired two-tailed *t*-test, ***p < 0.001.

Cardiomyocytes derived from both long QT syndrome lines were quantified using flow cytometry with MF20 labeling and found to be >80% pure [Fig. 4(a)]. Cardiomyocyte maturation was measured by cell proliferation with Ki67 labeling and morphological changes by *α*-actinin labeling (Fig. 4). Cardiomyocytes derived from both long QT lines exhibited increasingly more mature phenotypes over 150 days of culture, including *α*-actinin organization within sarcomeres [Fig. 4(b), day 150]. Cardiomyocytes derived from iPSC UCSD 106i-2–5 exhibited a relatively higher proliferation rate on day 20 [20.3% Ki67, Fig. 4(a)] and remained highly proliferative on day 40 [16.7% Ki67, Fig. 4(a)], whereas cardiomyocytes derived from UCSD 102i-2–1 line only had ~10.5% and 8.9% Ki67 populations on days 20 and 40, respectively. Cardiomyocytes derived from both iPSC lines showed increases in cell surface area, cell perimeter, the ratio of long versus short cell axes, and sarcomere length during extended culture *in vitro* for 150 days [Figs. 4(b) and 4(c), Fig. S4 in the Supplementary Material), consistent with the maturation trends seen in H9-derived CMs.

**Fig. 5:**
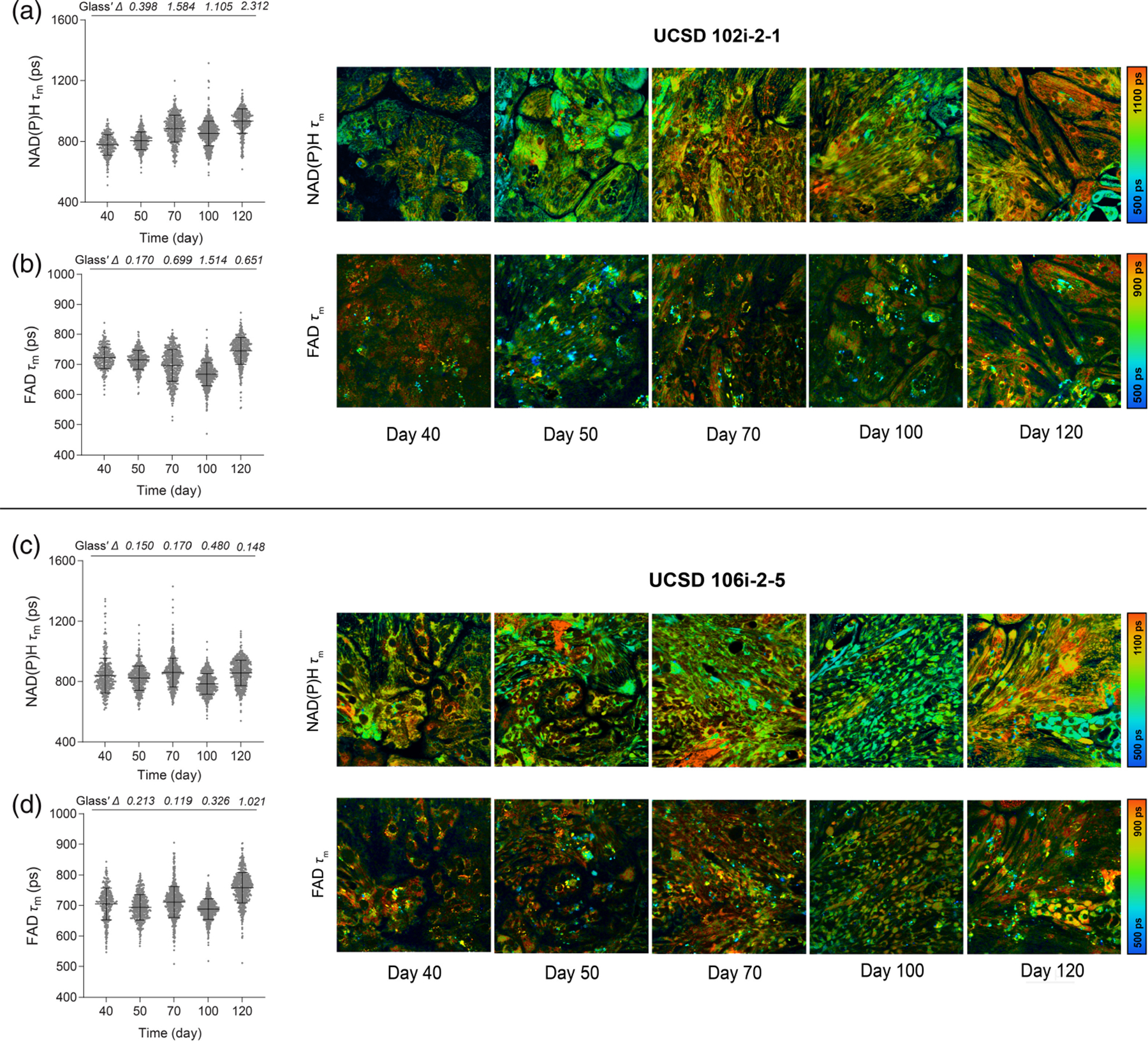
OMI detects varying metabolic phenotypes in cardiomyocytes with long QT syndrome during long-term *in vitro* maturation. Multiphoton autofluorescence OMI was performed on long QT iPSC-CMs throughout extended culture maturation beginning at day 40. Single-cell cytoplasmic quantitative analysis of UCSD102i (a) NAD(P)H mean lifetime (*τ*_*m*_) and (b) FAD mean lifetime (*τ*_*m*_) and of UCSD106i (c) NAD(P)H *τ*_*m*_ and (d) FAD *τ*_*m*_ are shown with corresponding representative images. Cells were measured on days 40, 50, 70, 100, and 120 (UCSD102i *N* = 271, 398, 483, 617, 431 cells, by day, 10 fields of view from three independent repeats; UCSD106i *N* = 337, 473, 649, 677, 654 cells, by day, 10 fields of view from three independent repeats). Data are presented as mean ± SD. The effect size was determined using Glass’ Δ with >0.80 considered a very large effect.

To monitor metabolic maturation in long QT iPSC-derived cardiomyocytes, we then performed OMI on cardiomyocytes derived from both iPSC lines on days 40, 50, 70, 100, and 120. We extended our maturation monitoring period to 120 days to allow sufficient time to observe changes in OMI variables that resembled those seen in H9-derived CMs. All eight autofluorescent variables were collected from the cytoplasm of individual cells. NAD(P)H *τ*_*m*_ in cardiomyocytes derived from UCSD 102i-2–1 increased gradually from day 40 to day 120, similar to the H9-CMs [Fig. 5(a)]. NAD(P)H *τ*_1_, *τ*_2_, and *α*_2_ also gradually increased from days 40 to 120, driving the observed mean lifetime changes [Fig. S5(a) in the Supplementary Material]. The FAD *τ*_*m*_ of UCSD 102i-2–1 also increased by day 120 relative to day 40 [Fig. 5(b)], mostly due to increases in FAD *τ*_2_ [Fig. S6(a) in the Supplementary Material]. Interestingly, it slightly decreased on days 70 and 100 relative to early maturation, similar to the trend observed in embryonic (H9) CMs. Conversely, NAD(P)H *τ*_*m*_, *τ*_2_, and *α*_2_ did not show an obvious increase in cardiomyocytes derived from UCSD 106i-2–5 throughout the maturation period [Fig. 5(c), Fig. S5(b) in the Supplementary Material]. Free NAD(P)H (*τ*_1_) increased in cells by day 120 [Fig. S5(b) in the Supplementary Material]. The FAD *τ*_*m*_ fluctuated minimally over time in cardiomyocytes derived from UCSD 106i-2–5 [Fig. 5(d)], whereas FAD *τ*_1_ and *τ*_2_ increased on day 120 compared with day 40 [Fig. S6(b) in the Supplementary Material]. This indicates that OMI may be sensitive to variability in the metabolic time course of maturation across different patient-derived iPSC lines.^72,73^

**Fig. 6:**
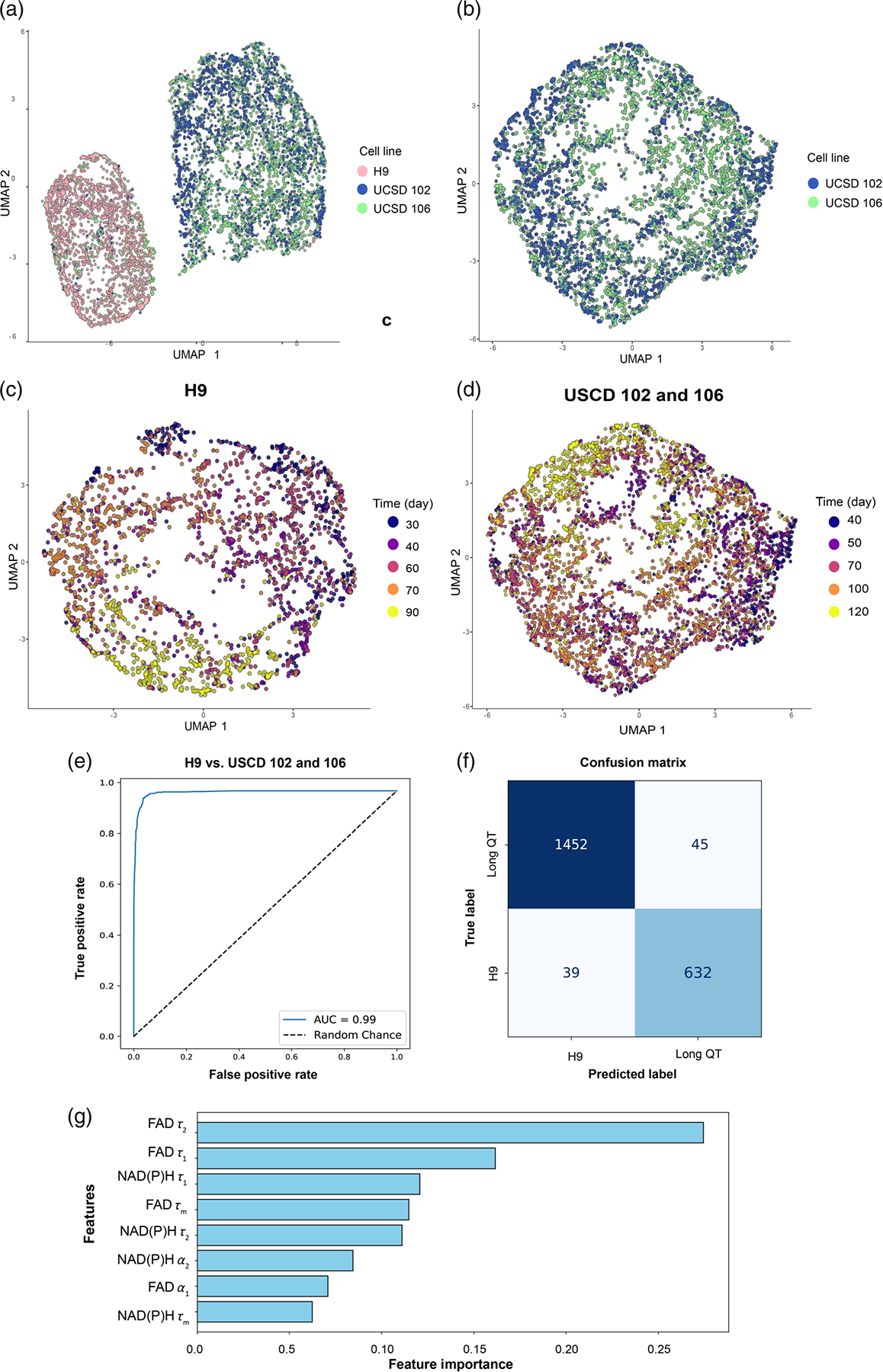
Multivariate analysis reveals unique metabolic profiles in stem cell–derived cardiomyocytes. UMAP dimensionality reduction was performed using eight autofluorescent variables (NAD(P)H *τ*_*m*_ *τ*_1_, *τ*_2_, *α*_1_; FAD *τ*_*m*_ *τ*_1_, *τ*_2_, *α*_1_) and projected onto two-dimensional space. (a) Color coding by cell type reveals the H9 (healthy ESC-derived) and long QT (iPSC-derived) cells cluster separately. (b) Color coding of long QT cells by line indicates some level of metabolic similarity among donors. (c) H9-derived CMs do not initially cluster (day 30) but clearly separate throughout late maturation (days 70 to 90). (d) Long QT lines follow a similar trajectory, with less clustering during early maturation but clearer separation by day 120. (e)–(g) A random forest classifier was used to determine whether the eight OMI variables can classify H9 from UCSD-102i-2–1 and UCSD-106i-2–5 cells with data pooled across all time points. (e) The AUC of the ROC for this binary classifier is 0.99, indicating high sensitivity. A total of 70% of the cells were used as the training set, and 30% of the cells were used as the validation set (*N* = 2235 H9 cells; *N* = 4990 long QT cells). (f) The confusion matrix of the test indicates high classification accuracy (96.13%) of healthy versus diseased cells. (g) OMI variable feature importance for the random forest classifier.

Next, to assess the differences in the metabolic time course across all three lines of hPSC-CMs, we performed UMAP dimensionality reduction on eight OMI variables (NAD(P)H *τ*_*m*_ *τ*_1_, *τ*_2_, and *α*_1_ and FAD *τ*_*m*_ *τ*_1_, *τ*_2_, and *α*_1_) across all time points. UMAPs reveal that H9 ESC-CMs cluster separately from both long QT iPSC lines, indicating clear metabolic differences across CMs derived from healthy embryonic and diseased iPSC lines [Fig. 6(a)]. UCSD-102i-2–1 and UCSD-106i-2–5 display some overlap, and their cluster densities are differently distributed, suggesting metabolic similarity among the lines throughout most of the time course despite significantly different levels of heterogeneity [Fig. 6(b)].

Due to the clear separation between normal embryonic and diseased iPSC CM lines, we separately assessed clustering at different time points. Clear clustering is not present during early maturation (day 30) in the H9-CM timeline; however, more separation is apparent as cells continue to mature, with clusters visible by day 70 [Fig. 6(c)]. Similarly, the UCSD-102i-2–1 and UCSD-106i-2–5 cells do not clearly separate until day 120, late in the maturation timeline [Fig. 6(d)].

Given this separation between human embryonic-derived and long QT–derived iPSC lines, we tested whether a machine learning classifier could distinguish these cell types. Specifically, we evaluated whether a random forest classifier based on the eight OMI variables could separate H9 cells from UCSD-102i-2–1 and UCSD-106i-2–5 cells with data pooled across all time points (binary classifier). The AUC of the ROC for this classifier is 0.99, indicating high sensitivity and specificity for discriminating H9 cells from UCSD-102i-2–1 and UCSD-106i-2–5 cells across all time points [Fig. 6(e)]. The confusion matrix similarly indicates high classification accuracy [Fig. 6(f)] of 96.13%. The feature importance of this random forest classifier indicates that FAD *τ*_2_ and *τ*_1_ are the most important variables for the separation of these cell types.

## 4 Discussion

Here, we report on a noninvasive imaging technique capable of monitoring metabolic changes in hPSC-CMs during long-term culture. We monitored cardiomyocytes as they matured for 90+ days using OMI and evaluated metabolic imaging parameters at the single-cell level. Our analyses revealed that OMI is sensitive to metabolic changes occurring during maturation under different conditions, including varying substrate stiffnesses and iPSC-derived cardiomyocytes carrying long QT disease phenotypes. We observed changes in NAD(P)H and FAD lifetimes in hPSC-CMs (Fig. 2) and identified subpopulations of cells based on single-cell analysis of NAD(P)H and FAD *τ*_*m*_ (Fig. S2 in the Supplementary Material). This heterogeneity may reflect distinct cell lineages and maturation trajectories of CMs in culture as not all cells mature in synchrony.^27–29^ The population separation observed in Fig. 6(a) between CMs derived from H9-CMs and long QT-CMs could be influenced by differences in donors’ age. Though we monitored all cell lines and overlapping time points (days 40 and 70), H9-CMs and long QT-CMs cluster in the UMAP space, as shown in Fig. 6(a).^74,75^ However, OMI is sensitive to differences in the metabolic time course among iPSC-CMs derived from two different donors with long QT type 2, indicating patient-level metabolic heterogeneity (Fig. 5). We further demonstrated that OMI can be used to monitor the impact of other maturation strategies (e.g., tuning the culture substrate stiffness). Future studies should incorporate additional validation methods, such as metabolic flux assays and RNA sequencing to further assess the robustness of OMI as a noninvasive tool for monitoring CM maturation. Overall, these results suggest that the noninvasive and single-cell imaging capabilities of OMI can provide unique insights into metabolic changes in hPSC-CMs during long-term culture, with applications in disease modeling and regenerative therapy.

Characterization of hPSC-CMs is typically accomplished using highly sensitive analyses such as immunostaining for expression of specific structural proteins and patch clamp measurements or calcium imaging for contractile assessment.^23^ Although certain techniques can be applied to single cells (e.g., patch clamp physiology, single-cell RNA sequencing), these are low throughput and/or endpoint measurements, requiring the cells to be fixed or destroyed. This impedes the ability to assess CM maturation as it progresses at the single-cell level. As demonstrated here, OMI can be repeatedly performed on the same cells as they mature, capturing the significant metabolic shift that individual cardiomyocytes undergo during fetal development as they transition from reliance on glycolysis to mitochondrial oxidative phosphorylation. This switch alters the binding properties of the autofluorescent metabolic coenzymes NAD(P)H and FAD. Using OMI, we found that NAD(P)H *τ*_*m*_ *τ*_1_, *τ*_2_, and *α*_2_ gradually increased during the maturation of healthy ESC-CMs (Fig. 2, Fig. S1 in the Supplementary Material), indicating increased protein-binding activities and changes in preferred NAD(P)H binding partners as expected.^76,77^

Although OMI reveals single-cell heterogeneity and reflects changes in the local environment, it cannot directly determine which specific molecules NAD(P)H and FAD interact with. Furthermore, OMI reports only on the metabolic facet of CM maturation. Therefore, complementary measurements of structure and function are needed to fully characterize stem cell–derived CMs as they mature and assess the influence of disease state or environmental manipulation. Importantly, the noninvasive nature of OMI allows other analyses such as flow cytometry or immunostaining to be performed on the same cell batches. In our studies, we observed that metabolic changes detected with OMI accompanied other facets of cardiomyocyte maturation, namely, expected structural changes [e.g., increased sarcomere size and alignment, Figs. 1(b) and 1(c)] and cell cycle changes [decreased proliferation, Fig. 1(a)].^8^ Our OMI measurements are therefore consistent with expected cellular structural changes and proliferative ability. H9-derived and UCSD102i-2–1-derived CMs showed similar changes in proliferation [Figs. 1(a) and 4(a)] and NAD(P)H metabolism [Figs. 2(a) and 5(a)] compared with UCSD106i-2–5-derived CMs [Figs. 4(a) and 5(c)]. The noninvasive nature of OMI further allows micro-environmental influences on cardiomyocyte maturation to be monitored over time.

Cardiomyocyte maturation is influenced in part by the mechanical micro-environment,^58^ with different synthetic two-dimensional and three-dimensional culture systems being employed *in vitro* to accelerate maturation.^78^ We observed metabolic changes induced by PDMS substrates at 15 kPa during early maturation, characterized by an increase in NAD(P)H *τ*_m_ and a decrease in FAD *τ*_m_ (Fig. 3). These changes resemble the maturation process of H9-CMs measured by OMI, which shows an increase in NAD(P)H *τ*_m_ from days 30 to 90 and a decrease in FAD *τ*_m_ from days 40 to 70 [Figs. 2(a) and 2(b)]. The similar changes observed by OMI—occurring only in CMs on 15 kPa substrates at an early stage and in H9-CMs at a later stage, but not on other substrates—may be attributed to the mechanical properties of 15 kPa, which closely resemble those of healthy cardiac tissue. This optimal mechanical micro-environment likely accelerates the maturation process.^79^ Overall, we demonstrated the ability to repeatedly image the same cell batches on various surfaces, indicating that OMI could be used to monitor the effects of environmental manipulation on maturing cardiomyocytes. Label-free imaging techniques could also be useful for assessing the metabolic effects of chemical perturbations, electrical and mechanical stimulation, and coculturing with other cell types.^47,78^

The metabolic trajectory of maturing cardiomyocytes can be affected by disease states, where abnormal cellular metabolism is thought to be responsible in part for CVDs.^80,81^ The physiologic consequences of long QT syndrome, for example, are poorly understood, and the phenotype of these CMs may be due in part to an atypical maturation process. OMI measurements were sensitive to donor-level heterogeneity, with NAD(P)H *τ*_*m*_ and protein binding increasing in the UCSD-102i-2–1 line over 120 days but not the USCD-106i-2–5 cells. However, FAD *τ*_*m*_ increased at the latest time point for both H9 (day 90) and USCD-106i-2–5 cells (day 120). The differences between the two LQT2 patients indicate increased oxidative metabolism in the USCD-102i-2–1 line relative to its counterpart (Fig. S5 in the Supplementary Material).^76^ This variation may be partially attributed to the different ages of parental cells, as well as multiple factors contributing to the signals in the FAD channel, such as lipids, as indicated by the punctate morphology observed in the representative images.^49,82^ The analysis of metabolism in healthy H9-derived cardiomyocytes and long QT–derived cardiomyocytes was complicated by their distinct metabolic trajectories over the maturation time. However, UMAP projections of healthy (H9) and diseased (long QT) lines over time showed clustering based on OMI variables at later time points, with more distinct separation occurring during later maturation. Future analyses should assess metabolic differences among cardiomyocytes with distinct forms of long QT syndrome. OMI could also be used to investigate patient-level differences in other CVDs.

## 5 Conclusions

hPSCs hold significant potential in advancing regenerative medicine and drug development. However, large-scale cell manufacturing from hPSCs lacks consistency and real-time quality control. Although hPSC-CMs exhibit some properties of mature cardiomyocytes through extended culture or external stimulation (e.g., mechanical, electrical),^83^ their functionality significantly differs from their *in vivo* counterparts.^84^ We have demonstrated that OMI is a powerful single-cell imaging tool capable of monitoring cardiomyocyte metabolism over long-term culture, thereby accelerating method development to enhance maturation. By tracking the autofluorescence information of NAD(P)H and FAD, this imaging technology can noninvasively study cellular behaviors related to metabolism, including mechanistic studies for diseases, toxicity testing, and drug development. OMI can also be used to complement other functional assays to provide a clearer picture of CM maturation and a greater understanding of CVD mechanisms. Such label-free imaging technologies may eventually be applied to drug discovery and cell manufacturing with the goal of providing quality control, increasing yield, and lowering cost.

## Supplementary Material

supplemental figures

## Statement of Discovery

We demonstrate that label-free optical metabolic imaging can noninvasively monitor single-cell metabolic changes during long-term culture of stem-cell–derived cardiomyocytes.
