# Supplementary material for: Label-free method to monitor metabolism during long-term culture of human pluripotent stem cell derived cardiomyocytes
Source: Biophotonics Discov. 2025 Apr 17;2(2):025001. doi: 10.1117/1.BIOS.2.2.025001 (PMC12225635; doi:10.1117/1.BIOS.2.2.025001)
Supplement: Supplementary file 1 [file BIOS_002_025001_SD001.pdf]

Supplemental Figures for BIOS-250064GR

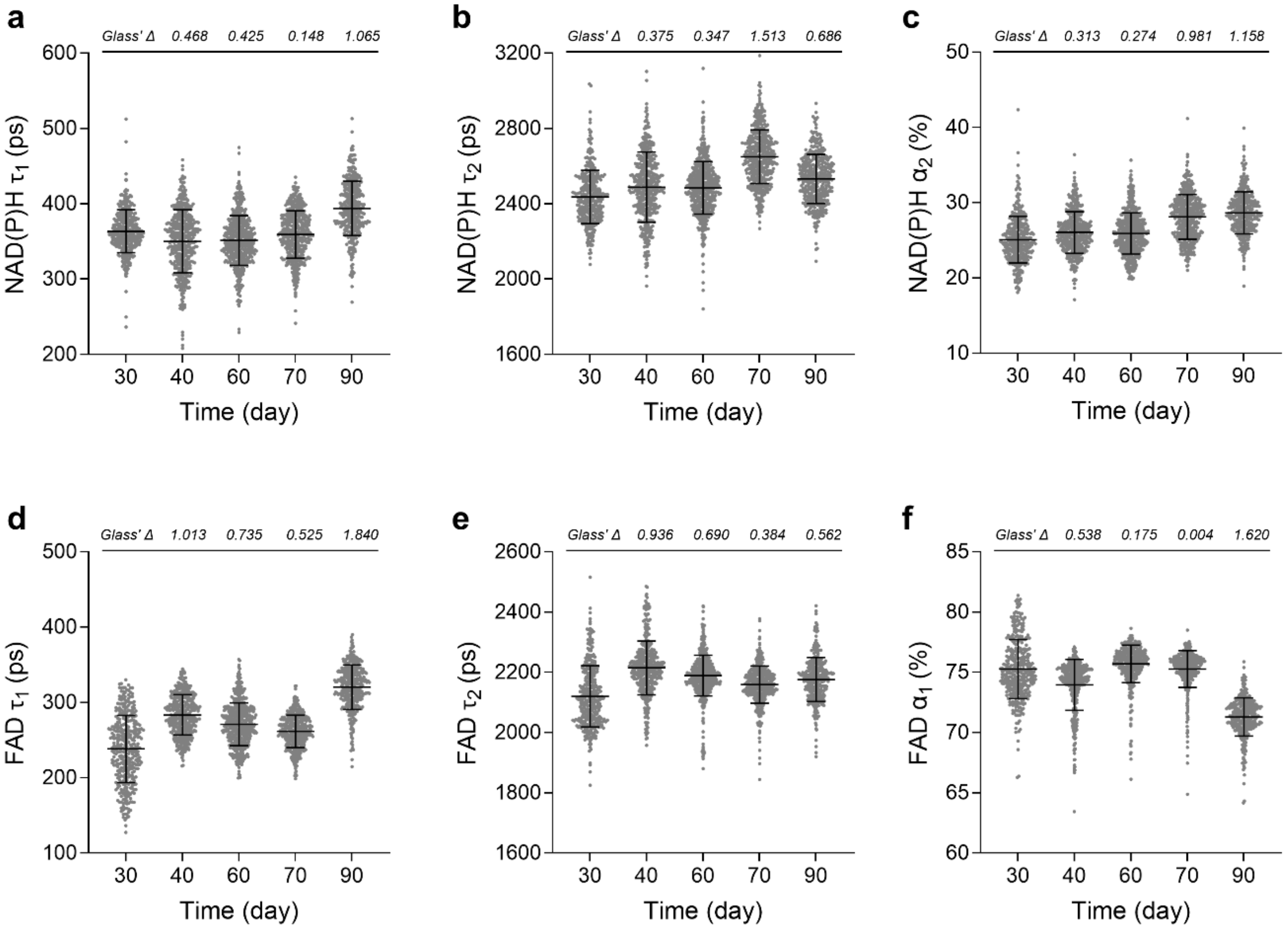

Fig. S1 OMI detects oxidative metabolic phenotype during long-term in vitro maturation. Multiphoton autofluorescence FLIM was performed on H9 ESC-derived cardiomyocytes throughout extended culture maturation from day 30 to day 90. Single-cell cytoplasmic quantitative analysis of **a-c)** NAD(P)H τ_1, τ_2, α_2, and **d-f)** FAD τ_1, τ_2, α_1, measured on days 30, 40, 60, 70, and 90 (*N* = 415, 485, 582, 465, 380 cells, respectively, collected across three independent repeats). Data are presented as mean ± SD. Effect size was determined using Glass' Δ with 0.5-0.8 considered a large effect and > 0.80 considered a very large effect.

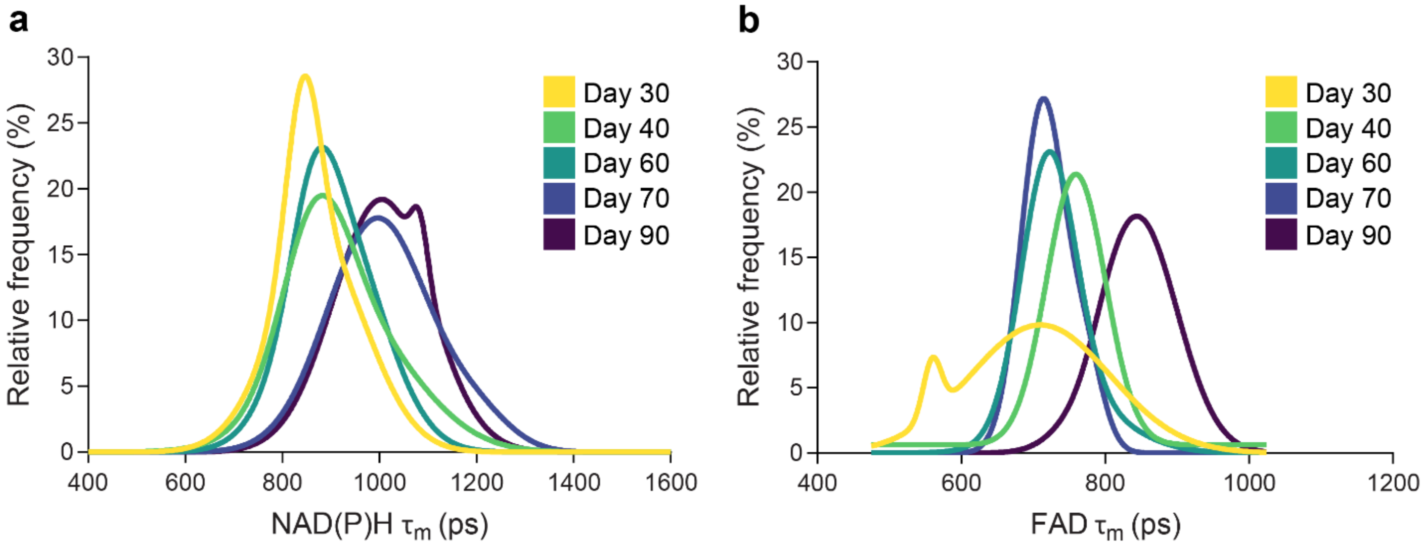

Fig. S2 OMI is sensitive to cardiomyocyte metabolic subpopulations during long-term maturation. H9-CM FLIM was performed on days 30, 40, 60, 70, and 90 (N = 415, 485, 582, 465, 380 cells, respectively, collected across three independent repeats). Single-cell cytoplasmic quantitative analysis of **a)** NAD(P)H τ_m shows a consistent, gradual increase throughout extended culture with longer lifetime subpopulations appearing in late maturation (day 90). **b)** Single-cell FAD τ_m analysis reveals short lifetime subpopulations that disappear after early maturation (day 30) and likely reveal the presence of broadly emitting lipids in addition to detected FAD signal.

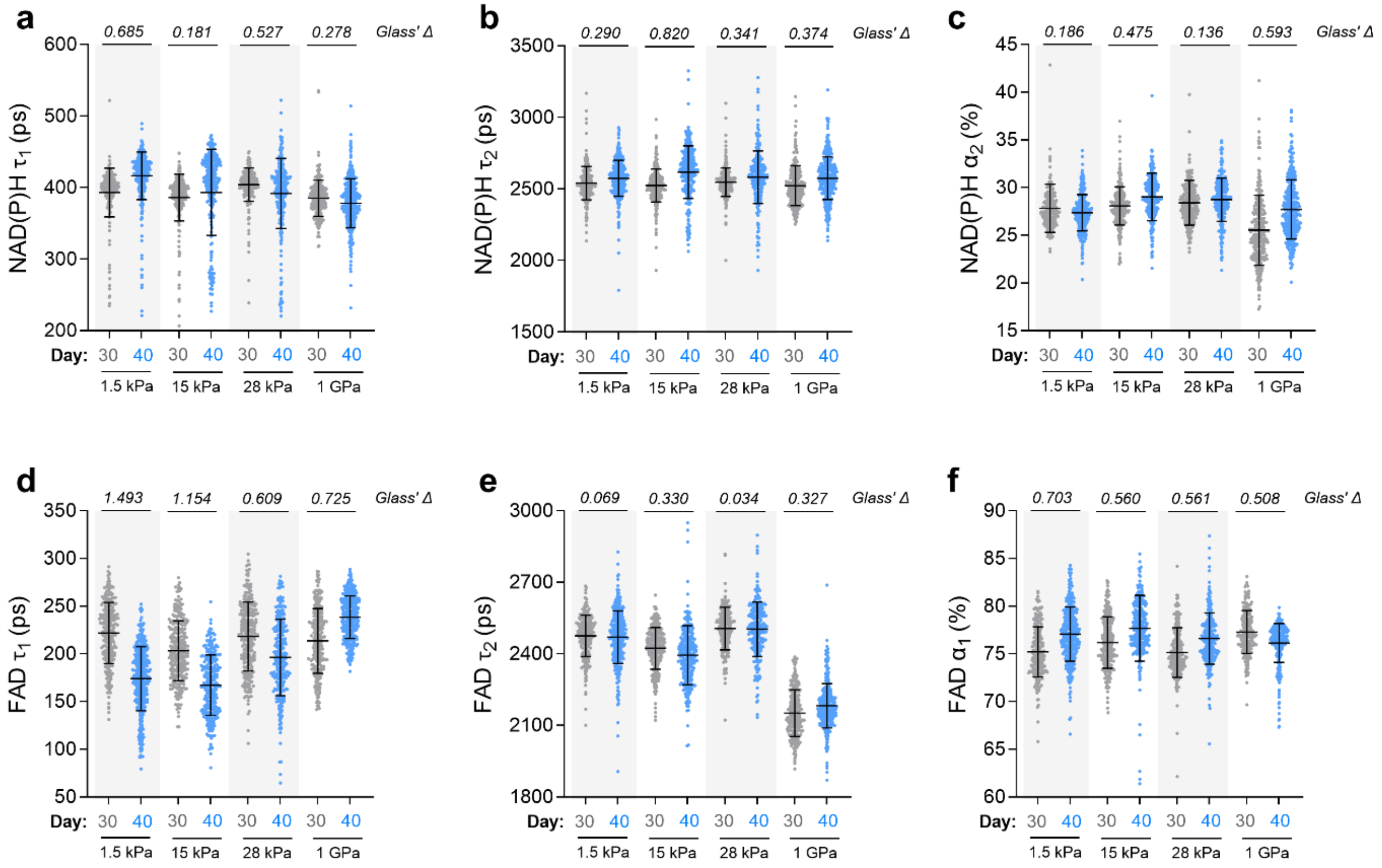

Fig. S3 OMI detects differences caused by substrate stiffness during maturation. H9 ESC-CMs were cultured on Matrigel-coated PDMS surfaces with different stiffnesses (1.5 kPa, 15 kPa, and 28 kPa) or Matrigel-coated polymer-bottomed dishes (1 GPa) and multiphoton FLIM was performed during early maturation (days 30 and 40). Single-cell cytoplasmic quantitative analysis of **a-c)** NAD(P)H τ_1, τ_2, α_2, and **d-f)** FAD τ_1, τ_2, α_1, was measured on day 30 (N = 271, 289, 269, and 274 cells, with increasing stiffness) and day 40 (N = 395, 295, 267, and 375 cells, with increasing stiffness). Data are presented as mean ± SD. Effect size was determined using Glass' Δ with > 0.80 considered a very large effect.

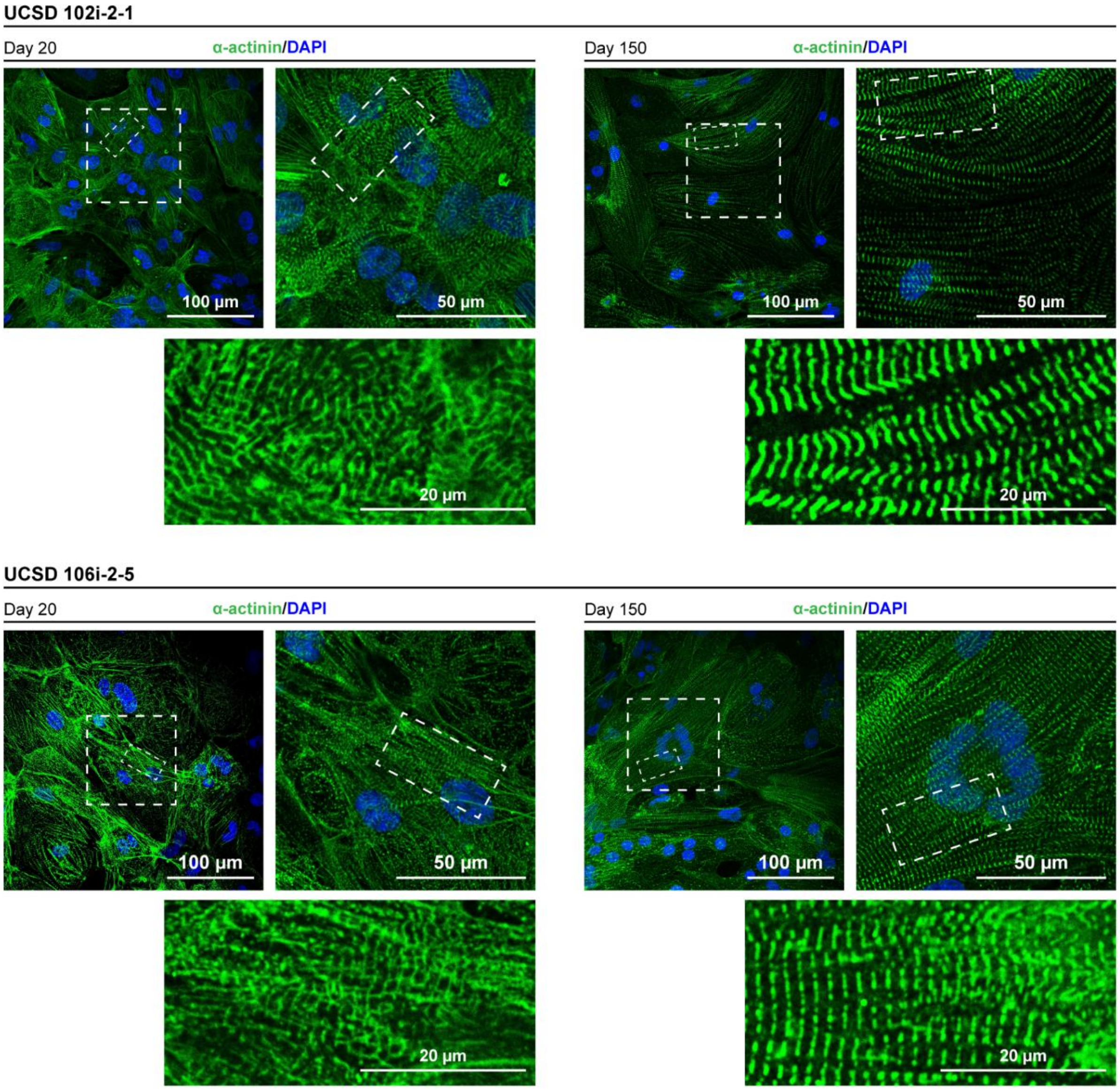

Fig. S4 iPSC-derived long QT cardiomyocytes exhibit a mature phenotype with extended in vitro culture. Human iPSCs carrying a long QT syndrome genetic deficiency were differentiated into long QT cardiomyocytes (UCSD102i-2-1, UCSD106i-2-5) following an established method[12]. Representative immunofluorescent α-actinin staining on day 20 shows sarcomere development, with more developed sarcomere organization present by day 150.

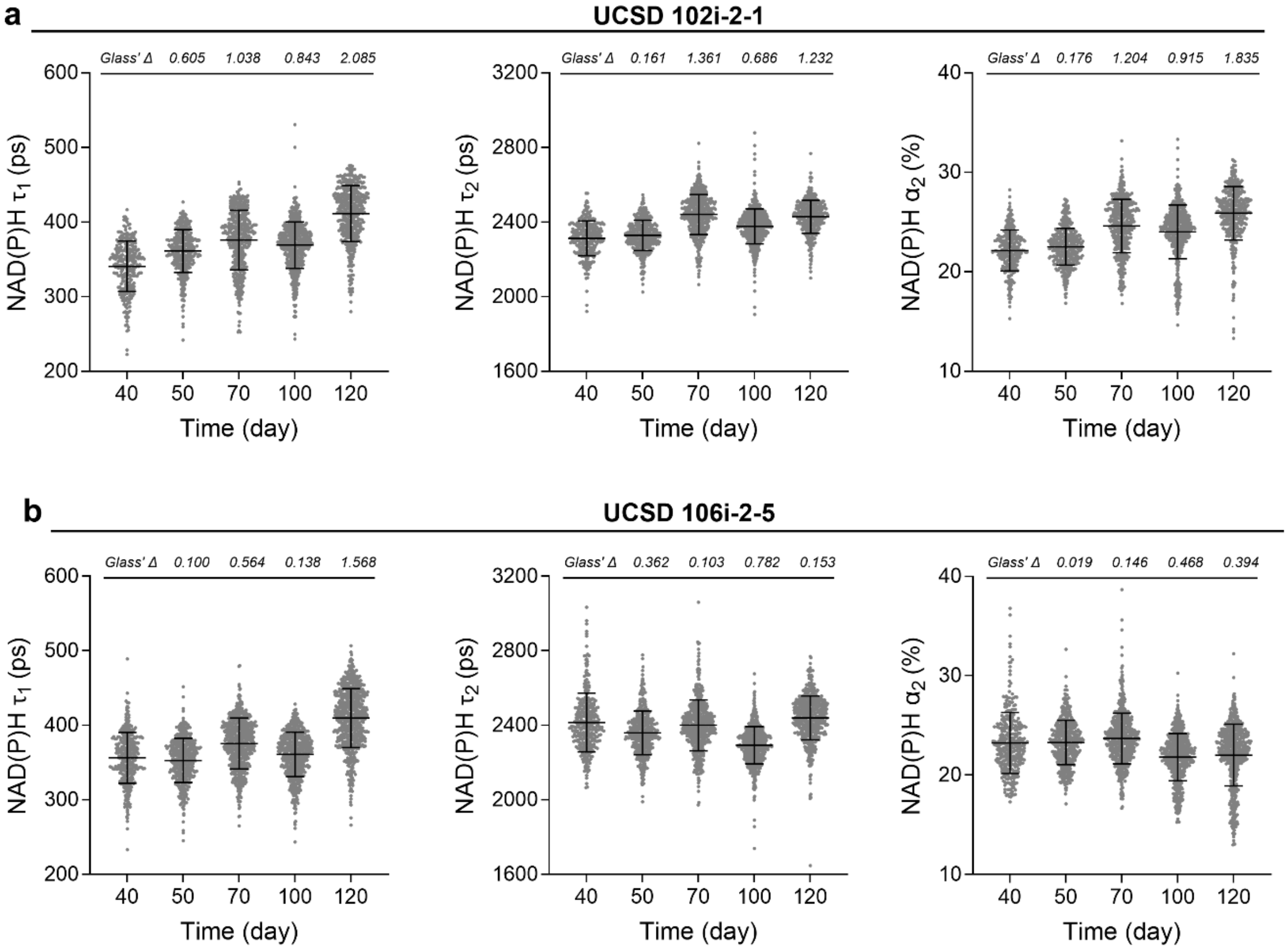

Fig. S5 OMI detects varying metabolic phenotypes in cardiomyocytes with long QT syndrome during long-term in vitro maturation. Multiphoton autofluorescence FLIM was performed on long QT iPSC-CMs throughout maturation beginning at day 40. Single-cell cytoplasmic quantitative analysis of NAD(P)H τ_1, τ_2, α_2, for **a)** UCSD 102-i-2-1 and **b)** UCSD 106-i-2-5 was measured on days 40, 50, 70, 100, and 120, and reveals patient-level heterogeneity in OMI fit parameters. UCSD102i-2-1 (N = 271, 398, 483, 617, 431 cells, by day) and UCSD106i-2-5 (N = 337, 473, 649, 677, 654 cells, by day). Data are presented as mean ± SD. Effect size was determined using Glass' Δ with 0.5-0.8 considered a larger effect and > 0.80 considered a very large effect.

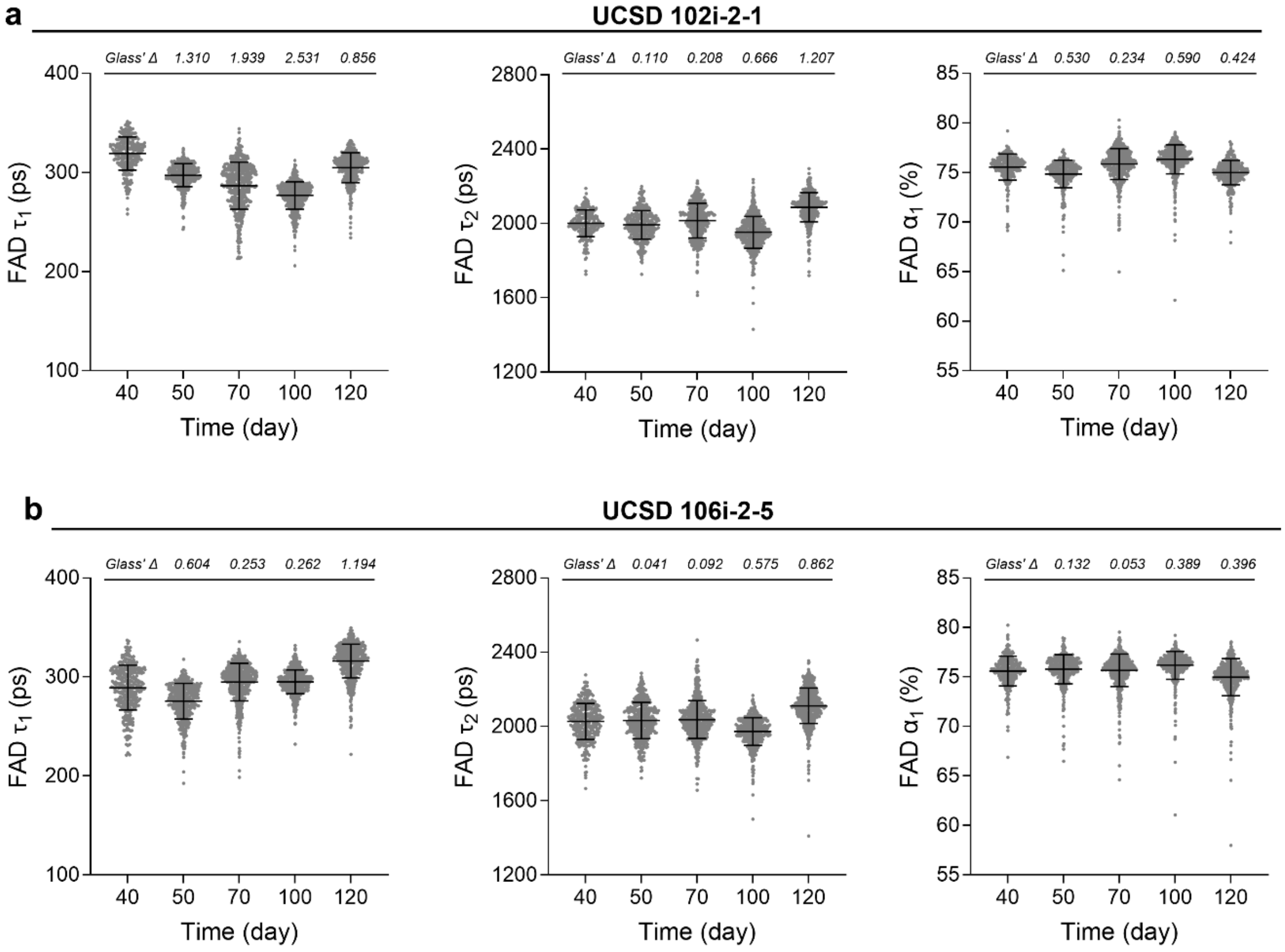

Fig. S6 OMI detects changes in FAD metabolism in cardiomyocytes with long QT syndrome during long-term in vitro maturation. Multiphoton autofluorescence FLIM was performed on long QT iPSC-CMs throughout maturation beginning at day 40. Single-cell cytoplasmic quantitative analysis of FAD τ_1, τ_2, α_1, for **a)** UCSD 102-i-2-1 and **b)** UCSD 106-i-2-5 was measured on days 40, 50, 70, 100, and 120, and reveals patient-level heterogeneity in OMI fit parameters. UCSD102i-2-1 (*N* = 271, 398, 483, 617, 431 cells, by day) and UCSD106i-2-5 (*N* = 337, 473, 649, 677, 654 cells, by day). Data are presented as mean ± SD. Effect size was determined using Glass' Δ with 0.5-0.8 considered a large effect and > 0.80 considered a large effect.
